# Validation of a case definition for depression in administrative data against primary chart data as a reference standard

**DOI:** 10.1186/s12888-018-1990-6

**Published:** 2019-01-07

**Authors:** Chelsea Doktorchik, Scott Patten, Cathy Eastwood, Mingkai Peng, Guanmin Chen, Cynthia A. Beck, Nathalie Jetté, Tyler Williamson, Hude Quan

**Affiliations:** 1 Department of Community Health Sciences, Cumming School of Medicine, University of Calgary, TRW Building 3rd Floor 3280 Hospital Drive NW, Calgary, AB T2N 4Z6 Canada; 2 Department of Psychiatry, Cumming School of Medicine, University of Calgary, 1403-29 Street NW, Calgary, AB T2N 2T9 Canada; 3 Department of Clinical Neurosciences, Cumming School of Medicine, University of Calgary, 1403 29 Street NW, Calgary, AB T2N 2T9 Canada

**Keywords:** Depression, Depressive disorder, Surveillance, Case definitions, Administrative data, International disease classification, Health information

## Abstract

**Background:**

Because the collection of mental health information through interviews is expensive and time consuming, interest in using population-based administrative health data to conduct research on depression has increased. However, there is concern that misclassification of disease diagnosis in the underlying data might bias the results. Our objective was to determine the validity of International Classification of Disease (ICD)-9 and ICD-10 administrative health data case definitions for depression using review of family physician (FP) charts as the reference standard.

**Methods:**

Trained chart reviewers reviewed 3362 randomly selected charts from years 2001 and 2004 at 64 FP clinics in Alberta (AB) and British Columbia (BC), Canada. Depression was defined as presence of either: 1) documentation of major depressive episode, or 2) documentation of specific antidepressant medication prescription plus recorded depressed mood. The charts were linked to administrative data (hospital discharge abstracts and physician claims data) using personal health numbers. Validity indices were estimated for six administrative data definitions of depression using three years of administrative data.

**Results:**

Depression prevalence by chart review was 15.9–19.2% depending on year, region, and province. An ICD administrative data definition of ‘2 depression claims with depression ICD codes within a one-year window OR 1 discharge abstract data (DAD) depression diagnosis’ had the highest overall validity, with estimates being 61.4% for sensitivity, 94.3% for specificity, 69.7% for positive predictive value, and 92.0% for negative predictive value. Stratification of the validity parameters for this case definition showed that sensitivity was fairly consistent across groups, however the positive predictive value was significantly higher in 2004 data compared to 2001 data (78.8 and 59.6%, respectively), and in AB data compared to BC data (79.8 and 61.7%, respectively).

**Conclusions:**

Sensitivity of the case definition is often moderate, and specificity is often high, possibly due to undercoding of depression. Limitations to this study include the use of FP charts data as the reference standard, given the potential for missed or incorrect depression diagnoses. These results suggest that that administrative data can be used as a source of information for both research and surveillance purposes, while remaining aware of these limitations.

## Background

Depression is a mood disorder, with symptoms such as sadness, fatigue, loss of interest, and loss of appetite [1]. In Canada, the one-year prevalence of Major Depressive Disorder (a common form of depression) was 3.9% [2]. Currently, detection of depression in primary care in Canada is low [3]. One report found that sensitivity of detection by primary care physicians is 50.1%, and specificity is 81.3% [3]. Sensitivity of detection has been reported to be as low as 36.4% among non-psychiatric physicians [4]. Further, physicians only record a diagnosis of depression in 17.6–33.6% of cases [5, 6], which likely reduces the detection of depression in primary care records and EMR data. Use of population-level surveillance data may improve detection, documentation, prevention, and management of depression in the Canadian population.

Administrative data include diagnostic and procedural codes obtained from encounters with the healthcare system, including physician visits, prescriptions, and surgeries/procedures [7]. Specifically, discharge abstract data includes coded data using the International Classification of Disease, version 10-Canadian version (ICD-10-CA), demographic information, and clinical information about patient hospital discharges [8]. Importantly, the coded portion of the data includes the main condition diagnosis, and any secondary conditions that were also treated during their inpatient stay. Physician claims data includes information on the physicians, service utilization and demographics of their patients, and physician payment information [8]. Administrative data provide population-level information that have been used as a surveillance tool for chronic diseases [9]. As these data are routinely collected, they provide a cost-effective and efficient method for chronic disease surveillance in the Canadian population [9]. However, there is a need to develop and validate case definition algorithms of depression in administrative data.

Various case definitions of chronic diseases, including hypertension [10], diabetes mellitus [11], chronic kidney disease [12], epilepsy [13], have been validated using Canadian administrative data. Various studies have assessed the quality of administrative databases in Canada, many of which are summarized in a scoping review by Hinds and colleagues (2017). However, this review found that few studies validated methods to identify mental illness in administrative data [14]. These previous studies show that the validity of case definition algorithms vary in administrative data, with sensitivities ranging from 19.4–99.3%, and specificities ranging from 84.2–97.2%. In a recent study using Canadian administrative data, case definitions for depression were found to be sub-optimal (with sensitivities ranging from 28.9–35.6%) [15]. This emphasizes the need to optimize case definitions for depression in administrative data, and also to assess their validity so that strengths and weaknesses of the case definitions can be accounted for in its applications.

This study aimed to validate and compare the accuracy of several case definitions for depression using administrative health data from two Canadian provinces, compared to a reference standard of family physician (FP) chart reviews. The case definitions were from the 9th and 10th versions of the International Classification of Disease (ICD). Further, this study aimed to test the variation in the validity of the optimal case definition by stratifying by region, time period (2001 and 2004), province and patient sex, age, and comorbidities.

## Methods

### Recruitment of FPs and selection of patients

The methods used for the selection and collection of FP charts have been previously described in detail elsewhere. [10].

### Chart data collection and defining depression

Five trained individuals extracted data from randomly selected patient charts at FP clinics. Eligible patient charts included patients ≥35 years of age, who were alive during the study years, living in the provinces of AB or BC during the 2-year period before the study years (2001 and 2004), and who had at least 2 visits to a FP physician during the study years [10]. Training of the chart reviewers consisted of reviewing ten charts together, and coming to consensus on whether the patient had depression or not based on the definition below. Reviewers extracted other patient information, including demographics, medications, and comorbidities. Comorbid conditions were defined by Quan et al. (2005), and included stroke, dementia, diabetes mellitus, dyslipidemia, coronary artery disease, peripheral vascular disease, congestive heart failure, chronic pulmonary disease, asthma, cancer, chronic kidney disease, hypertension, and dialysis [16].

Patients were defined as having depression if the charts stated either that (1) the patient had a Major Depressive Episode (MDE), OR (2) the patient was on antidepressants along with having clinic notes indicating a depressed mood. The antidepressants that were included were as follows: (1) Tricyclic Antidepressants, including amitriptyline, clomipramine, desipramine, doxepin, imipramine, nortriptyline, protriptyline, trimipramine, (2) Monoamine Oxidase Inhibitors, including isocarboxazid, phenelzine, and tranylcypromine; (3) Heterocyclics, including amoxapine, buproprion, maprotiline, and trazodone; (4) Selective Serotonin Reuptake Inhibitors, including fluoxetine, paroxetine, and sertraline; (5) Serotonin and Noradrenaline Reuptake Inhibitors, including duloxetine, and venlafaxine; and (6) Noradrenergic and Specific Serotonergic Antidepressants, including mirtazapine. Patient were coded as not having depression if any of the following were stated on the chart: (1) clinic notes indicated that the patient had only a depressed mood (rather than a diagnosis of MDE) but was not taking any of the previously listed medications; (2) patients with only a depressed mood (rather than a diagnosis of MDE) were taking a medication from this list, but it was clearly prescribed for a reason other than depression (e.g. for chronic pain, fibromyalgia, or neuropathic pain); (3) the patient was diagnosed with manic depression; or (4) the patient was diagnosed with bipolar disorder (i.e., manic depression).

### Defining depression using administrative data

Discharge abstract data, including main conditions, secondary conditions, and procedures, are recorded by Health Information Management (HIM) coding professionals and submitted to hospital administration and the Canadian Institute for Health Information (CIHI). Family practice physicians who are fee-for-service submit claims by documenting codes for patient conditions and procedures. These claims are submitted to the National Physician Database in Canada. To obtain administrative data, three databases were used (population registries, hospital discharge abstracts from AB between 1999 and 2004, and physician fee-for-service claims in AB and BC, both rural and urban, in both 2001 and 2004). We used discharge abstract data and claims data to capture both inpatient and outpatient points of service, to potentially enhance the validity of a case definition. The administrative data were linked to the FP office chart data using personal health numbers. The population registry database was used to obtain patient demographics, place of residence, death, and migration during the study period. This registry includes almost all AB and BC residents, as the Canadian healthcare insurance system is universal.

Discharge abstract data include inpatient discharges and deaths in AB and BC. ICD-9 CM codes were used for the years 1999, 2000, and 2001; ICD-10 codes were used in 2002, 2003, and 2004. ICD-9 CM and ICD-10 codes were used to identify patients with depression in the administrative data (ICD-9 CM codes: 296.2, 296.3, 296.5, 300.4, 309.x, and 311; ICD-10 codes: F20.4, F31.3-F31.5, F32.x, F33.x, F34.1, F41.2, and F43.2). These are the same ICD-9 CM and ICD-10 coding algorithms used to define depression as an Elixhauser comorbidity in administrative data by Quan and colleagues [16]. In the case of multiple diagnoses coded for a patient from the discharge summary (which becomes the discharge abstract database), we considered all diagnoses regardless of whether depression was the primary “main” condition, or a comorbid condition. The case definitions used to define depression in administrative data were as follows: (1) 1 physician claims within a three-year window (2) 2 physician claims within a 1 year window (3) 2 physician claims within a 2 year window (4) 2 physician claims within a 3 year window (5) 1 depression diagnosis from hospital discharge abstract data (DAD) and (6) 2 physician claims within a 1 year window or 1 DAD diagnosis.

### Statistical analysis

Demographic (age, sex, and region) and comorbidity variables were calculated and examined using descriptive statistics. For each of the six case definitions generated, each of the following parameters was calculated: sensitivity, specificity, positive predictive value (PPV), and negative predictive value (NPV). The chart data were used as a reference standard. For each parameter, a 95% confidence interval (CI) was calculated. These values were stratified by region, year, province, age, sex, and the presence of comorbidities.

## Results

### Characteristics of the study sample

A total of 3362 charts were reviewed at 64 FP clinics. The prevalence of depression in the sample based on chart review ranged from 15.9 to 19.2%, depending on the year (2001 or 2004), region (urban or rural), and province (AB or BC) (Table 1). The mean age of patients ranged from 52.2 to 54.2, and there were consistently more female (57.2–66.4%) than male (33.6–42.8%) patients. At least one of the assessed comorbidities was recorded for 35.3 to 44.6% of patients.

**Table 1:** Characteristics of Study Sample

	Year		Region		Province		Total
	2001	2004	Urban	Rural	AB	BC	
Number of patients	1482	1880	2372	990	1565	1797	3362
Age (mean ± SD)	53.2 ± 12.5	52.4 ± 12.8	52.2 ± 12.6	54.2 ± 12.7	52.3 ± 12.9	53.2 ± 12.5	52.8 ± 12.7
Sex (n, %)							
Male	506 (34.3)	711 (37.8)	796 (33.6)	424 (42.8)	641 (34.6)	679 (37.8)	1220 (36.3)
Female	973 (65.7)	1169 (62.2)	1576 (66.4)	566 (57.2)	1024 (65.4)	1118 (62.2)	2142 (63.7)
Comorbid Conditions (n, %)^a^							
Yes	523(35.29)	839(44.63)	962(40.56)	400(40.40)	614(39.23)	748(41.62)	1362(40.5)
Depression (n, %)							
Yes	236(15.92)	352(18.72)	402(16.95)	186(18.79)	301(19.23)	287(15.97)	588(17.49)

^a^Comorbidity includes stroke, dementia, diabetes mellitus, dyslipidemia, coronary artery disease, peripheral vascular disease, congestive heart failure, chronic pulmonary disease, asthma, cancer, hypertension, chronic kidney disease, and dialysis

### Determining a valid case definition for depression

The most valid case definition was 2 depression claims within a one-year period, or one DAD with a depression diagnosis (Table 2). The sensitivity was 61.4% (95% CI 57.3, 65.4%), the specificity was 94.3% (95% CI 93.4, 95.2%), the PPV was 69.7% (95% CI 65.5, 73.6%), and the NPV was 92.0% (95% CI 91.0, 93.0%). To determine if this was the most valid case definition, the sensitivity, specificity, PPV and NPV for various depression case definitions were assessed. Two billing claims were compared to one billing claim, and various time gaps were also evaluated. Two billing claims had consistently higher PPV compared to one billing claim, and therefore two billing claims were required as part of the case definition. Further, time gaps between claims were considered important in selecting a valid case definition. Collecting claims over longer periods of time is unfeasible in clinical practice, and changes in measures of validity after six months are negligible. Therefore, shorter time periods between claims were considered sensitive and clinically feasible for implementation. Further, a one-year time gap allows easier calculation of annual prevalence rate, a common period prevalence parameter in the depression literature. Two physician claims within one year was selected as the valid case definition, as it was clinically feasible to implement, and demonstrated high validity (particularly the PPV).

**Table 2:** Validity of Different Administrative Data Depression Case Definition Compared with Chart Data

Administrative Data Case Definition	Sensitivity % (95% CI)	Specificity % (95% CI)	PPV % (95% CI)	NPV % (95% CI)
1 depression claim within 3 years	78.9 (75.4, 82.1)	86.5 (85.2, 87.8)	55.4 (51.9, 58.8)	95.1 (94.2, 95.9)
2 depression claims within 1 year	60.7 (56.6, 64.7)	94.4 (93.5, 95.2)	69.7 (65.5, 73.7)	91.9 (90.8, 92.9)
2 depression claims within 2 years	62.9 (58.9, 66.8)	93.8 (92.8, 94.7)	68.3 (64.2, 72.2)	92.3 (91.2, 93.2)
2 depression claims within 3 years	63.4 (59.4, 67.3)	93.7 (92.8, 94.6)	68.2 (64.1, 72.1)	92.4 (91.3, 93.3)
1 depression diagnosis from hospital discharge data	5.3 (3.6, 7.4)	99.6 (99.3, 99.8)	75.6 (59.7, 87.6)	83.2 (81.9, 84.5)
2 depression claims within 1 year or 1 hospital discharge data diagnosis	61.4 (57.3, 65.4)	94.3 (93.4, 95.2)	69.7 (65.5, 73.6)	92.0 (91.0, 93.0)

Validity of the case definitions assessing two depression claims were similar, regardless of whether the claims were made within a one, two, or three year period. However, one depression claim within three years had a lower specificity and PPV compared to the definitions using two claims (86.5, 95% CI 85.2, 87.8%; and 55.4, 95% CI 51.9, 58.8% respectively), however the sensitivity was slightly higher in comparison (78.9, 95% CI 75.4, 82.1%). One DAD diagnosis had a very low sensitivity (5.3, 95% CI 3.6, 7.4%), but a high specificity and PPV (99.6, 95% CI 99.3, 99.8%; 75.6, 95% CI 59.7, 87.6% respectively), in comparison to the other the case definitions.

### Stratification of validity

The validity for the case definition of two depression claims within one year, or one DAD diagnosis was stratified according to region, year, province, sex, age, and the presence of one or more comorbidities (Table 3). Prevalence of depression in administrative data was compared to the prevalence in the FP office chart data. The prevalence estimate of depression appeared to be higher in chart data for almost all groups, with the exception of the year 2001 the province BC. The prevalence of depression in chart data contrasted to prevalence in administrative data, as the chart data showed that depression appeared higher in rural vs. urban populations (18.8% vs. 16.9%), higher in the year 2004 vs. 2001 (18.7% vs. 15.9%), in AB vs. BC (19.2% vs. 16.0%), and in those with comorbidities vs. those without comorbidities (18.2% vs. 17.0%).

**Table 3:** Validity for a Case Definition Stratified by Region, Year, Province, and Patient Characteristics^a^

	N	Prevalence Admin Data (%)	Prevalence Chart Data (%)	Sensitivity (%) (95% CI)	Specificity (%) (95% CI)	PPV (%) (95% CI)	NPV (%) (95% CI)
Region							
Rural	990	14.9	18.8	58.6 (51.2, 65.8)	95.2 (93.4, 96.5)	73.7 (65.8, 80.5)	90.9 (88.7, 92.7)
Urban	2372	15.6	16.9	62.7 (57.8, 67.4)	94.0 (92.9, 95.0)	68.1 (63.1, 72.8)	92.5 (91.3, 93.6)
Year							
2001	1482	16.5	15.9	61.9 (55.3, 69.1)	92.1 (90.4, 93.5)	59.6 (53.2, 65.8)	92.7 (91.1, 94.1)
2004	1880	14.5	18.7	61.1 (55.8. 66.2)	96.2 (95.1, 97.1)	78.8 (73.4, 83.5)	91.5 (90.0, 92.8)
Province							
AB	1565	14.6	19.2	60.5 (54.7, 66.0)	96.4 (95.2, 97.3)	79.8 (74.0, 84.8)	91.1 (89.4, 92.6)
BC	1797	16.1	16.0	62.4 (56.5, 68.0)	92.7 (91.2, 93.9)	61.7 (55.9, 67.4)	92.8 (91.4, 94.1)
Sex							
Female	2142	17.6	20.4	61.2 (56.5, 65.8)	93.6 (92.3, 94.7)	70.8 (66.0, 75.4)	90.4 (89.0, 91.8)
Male	1220	11.6	12.5	61.8 (53.6, 69.6)	95.6 (94.2, 96.8)	66.7 (58.2, 74.4)	94.6 (93.1, 95.9)
Age (years)							
<65	2709	17.0	19.1	61.4 (57.1, 65.6)	93.5 (92.4, 94.5)	69.0 (64.5, 73.2)	91.1 (89.9, 92.3)
≥ 65	653	8.7	10.7	61.4 (49.0, 72.8)	97.6 (96.0, 98.7)	75.4 (62.2, 85.9)	95.5 (93.5, 97.0)
Comorbid Presence^b^							
Yes	1362	15.1	18.2	58.9 (52.5, 65.1)	94.6 (93.1, 95.9)	70.9 (64.2, 77.0)	91.2 (89.4, 92.8)
No	2000	15.6	17.0	63.2 (57.9, 68.4)	94.2 (92.9, 95.2)	68.9 (63.5, 74.0)	92.6 (91.2, 93.8)

^a^Stratified analysis used the case definition “2 claims within 1 year or 1 hospital discharge data diagnosis” in 3- year administrative data

^b^Comorbidity includes stroke, dementia, diabetes mellitus, dyslipidemia, coronary artery disease, peripheral vascular disease, congestive heart failure, chronic pulmonary disease, asthma, cancer, hypertension, chronic kidney disease, and dialysis

## Discussion

The sensitivities of various administrative data definitions of depression in this study ranged from 5.3–78.9%, indicating a suboptimal ability for these case definitions to correctly classify those patients who have FP-chart defined depression in administrative data. The optimal case definition found in this study was “two depression claims within one year or one DAD depression diagnosis,” which had a moderate level of sensitivity at 61.4% (95% CI 57.3, 65.4%), and a high level of specificity at 94.3% (95% CI 93.4, 95.2%). Similar to the current study, Fiest et al. found that sensitivities of depression case definitions in their administrative data were low (ranging from 28.9–35.6%) [15]. We suspect that the difficulty in correctly identifying depression in healthcare has led to high misclassification and poor sensitivity when developing case definitions using administrative data.

The moderate level of sensitivity when using the case definition “two depression claims within one year or one DAD depression diagnosis” in administrative data may be due to issues of undercoding of depression by physicians in claims data, and the healthcare data captured by administrative data. Approximately 94% of physicians record only one code per claim [17]; we suspect that mental illnesses are coded less often when patients also present with comorbidities (e.g., when a patient presents with both diabetes and depression, physicians tend to code for diabetes only). This has shown to also be the case in DAD data, where coding validity decreases when another condition is present [18]. Coding of conditions in discharge abstracts increases when the patient’s condition is clinically important and complex [18]. Further, the issue of undercoding may be due to stigma associated with mental illness. Stigma associated with depression can lead to patients avoiding the mental health care system. This can be due to patients not believing that treatment will benefit them, a lack of knowledge about treatment of depression, lack of knowledge about accessing treatment, and believing that they will be prejudiced or discriminated against [19, 20]. Further, administrative data may not include enough sources of healthcare data to capture all the patients with depression. Including additional data sources should be used to improve sensitivity of administrative data, including prescription data, electronic medical records (e.g., Alberta Netcare), psychiatric specialist data, the National Ambulatory Care Reporting System (NACRS), mental health data from clinics, and private psychologist data.

The comparator group of this study (physician chart review) is our limitation and may also have issues of moderate sensitivity. This may be because depression is difficult to diagnose in clinical settings. In one primary care study, physicians correctly identify depression in 47.3% of actual cases, resulting in 50.1% sensitivity and 81.3% specificity [6]. Only 33.6% of these physicians correctly recorded that their patient had depression in their patient medical records [6]. A systematic review and meta-analysis supported this evidence, and found that the sensitivity of identification of depression by non-psychiatric physicians was only 36.4% (95% CI 27.9–44.8%) [4]. In primary care settings, variable detection of depression could arise from any of the following: 1) patients with a clearer presentation of symptoms are easier to detect; 2) those patients with more severe depression are more likely to be diagnosed compared to mild forms of depression; 3) consultation time for complex patients could compromise the accuracy of the diagnosis; and 4) a stronger relationship between the physician and patient, as well as 5) a physician with more experience in medicine, is associated with better detection of depression [6]. Suboptimal sensitivity in primary care, as well as incomplete documentation of depression in medical records, can reduce the sensitivity of case definitions in administrative data. To demonstrate these issues, Canuto and colleagues found that the agreement between psychiatrists and other physicians in diagnosing depression was 40% [21]. Agreement levels increased when the patients presented with severe depressive symptoms, and if they had a more open personality with lower levels of neuroticism. These personality types likely are more understanding of the importance of medical care and maintaining mental well-being [22]. These findings underscore the low agreement between physicians, and the low sensitivity of diagnosing depression in primary care settings [4]. Future research should focus on improving identification and documentation of depression in primary care settings.

In the present study, we found negligible differences in measures of validity between different time frames. For example, there were negligible differences in measures of validity between having one depression claim within six months, versus having one depression claim within three years. This may be due to the characteristics of the study sample *(*i.e., patients with depression). Individuals with depression have been shown to be frequent users of the healthcare system (e.g., general practitioners, emergency departments, and psychiatric specialists) [23, 24]. Further, patients with poor mental health are 1.70 times more likely to use the emergency department (95% CI 1.42, 2.02) [23]. This evidence was supported by Byrne et al., who discovered that patients with poorer mental health were frequent users of other healthcare services (not just the emergency department), and should be considered a vulnerable population that have greater healthcare service needs [24]. Depressed patients who are frequent users of the healthcare system will be captured in the first case definition (one claim within six months). Thus, we chose to eliminate case definitions that had six-month intervals, and chose only to evaluate one, two, and three year windows. Further, we chose a final case definition that was practical to implement in healthcare, as the difference in validity between time frames was negligible regardless. We selected the case definition of two depression claims within a one-year window with a DAD diagnosis, as a one-year time frame is easier to implement and calculate an annual prevalence rate.

To create an appropriate case definition for depression in administrative data, a depression diagnosis in DAD was included in some definitions. Including one DAD diagnosis in the case definition did not significantly increase the validity of the case definition. However, patients captured in DAD tend to have poorer health compared to those found in claims data, as DAD includes hospital inpatient discharges, as well as day surgery interventions [8]. These patients have a higher prevalence of mental illness including depression from previous diagnoses, compared to claims data, which typifies a population with more severe conditions. Using DAD alone to develop a depression case definition would result in suboptimal validity, as DAD underreports comorbidities such as depression [25]. It is also possible that DAD diagnoses alone are biased. For example, in the case of diabetes mellitus, many patients are managed in outpatient clinics, and the severity of disease can be vastly different when comparing inpatients and outpatients [11, 26]. Ultimately, including a depression DAD diagnosis in the case definition would not significantly impact the surveillance of depression, as the sensitivity is so low in this database. However, if this group of sicker patients were not captured by the proposed case definition, use of this definition in health services outcome research would underestimate risk factor associations. Thus, DAD is important to include in the case definition for administrative data to improve its application.

The current study provides a case definition for depression in administrative data with a moderate level of validity. However, limitations remain with using administrative data. The reference standard used in this study (i.e., physician chart reviews from primary care settings) itself has a moderate level of sensitivity, making it a suboptimal comparator. Further, the administrative data used for the current study include population registries, hospital discharge abstracts, and physician fee-for-service claims. The claims data were collected from specialist referrals, and the validity of the case definition is compared to physician chart reviews. Thus, this study compared data from two different sources, which can result in moderate sensitivity.

The optimal case definition identified from the current study can be used in future research, but should be interpreted with caution. Because the sensitivity and PPV of the optimal definition are similar to one another (61.4 and 69.7%, respectively), using the definition “two depression claims within one year or one DAD diagnosis” for surveillance purposes would provide a relatively accurate prediction of depression prevalence in the AB and BC populations. Awareness, improved diagnosis, and reduced stigma of depression may increase the estimated prevalence over time, despite the true number of depressed patients staying relatively stable. Thus, this case definition should be re-evaluated over time to ensure accurate monitoring and surveillance of depression. Further, when using this case definition for analytic studies, measures of association should be interpreted carefully. Misclassification when using the case definition “two depression claims within one year or one DAD diagnosis” results in moderate sensitivity, and therefore potentially moderate frequencies of false negative cases and low frequencies of true positive cases. For example, a moderate frequency of depressed patients will be classified as not having depression. In the case of analytic studies assessing risk factors for depression, this misclassification may be expected to bias estimates of association towards the null, assuming that the misclassification is nondifferential. In the case of analytic studies assessing outcomes of depression (e.g., mortality), the moderate frequency of false negative cases resulting from the case definition will misclassify depressed patients as not being depressed. This may cause the control group to appear sicker, and vice versa, diluting the measure of association found in the study, and understating the true measure of association that is representative of the population.

## Conclusion

Administrative data is primarily used for disease surveillance and reporting by the Canadian Institute for Health Information and is increasingly used in research. It is a source of data that offers national coverage of population-level data. By improving the quality of administrative data, researchers can readily use this source of data with a confidence in its accuracy, and disease/mortality surveillance can correctly reflect prevalence of disease and mortality rates for global comparison and monitoring.

Consistency when coding depression using ICD-9 CM and ICD-10 can improve the sensitivity of administrative data. Although this research was conducted using Canadian administrative data, other countries are able to carry out similar validation studies of chronic disease case definitions using large population-level surveillance data. For example, the United States has access to databases such as the Veterans Health Administration healthcare system. While this study provides an example of developing a case definition for depression, caution should be exercised when using this case definition for surveillance and analytic research purposes. Efforts should be made to improve the coding of depression in administrative data. The current study can set an example for future research in other regions or countries that have access to population-level healthcare surveillance databases.

The case definition “two depression claims within one year or one DAD diagnosis” can be used for depression in administrative data, and results in a moderate level of sensitivity with a high specificity. While this case definition can be used to identify depression from these administrative data sources in Alberta and British Columbia, they are limited to these data sources, and to the diagnosis of depression only. Validity will vary, depending on the administrative data source used in different contexts, and the disease that is being identified. Validity of unique data sources and different diseases should be assessed prior to using the data for research and administrative purposes.

## Abbreviations

AB: Alberta
BC: British Columbia
CI: Confidence Interval
DAD: Hospital Discharge Abstract Data
FP: Family Physician
ICD: International Classification of Disease
MDE: Major Depressive Episode
NACRS: National Ambulatory Care Reporting System
NPV: Negative Predictive Value
PPV: Positive Predictive Value
